# Challenges During Implementation of a Patient-Facing Mobile App for Surgical Rehabilitation: Feasibility Study

**DOI:** 10.2196/humanfactors.8096

**Published:** 2017-12-07

**Authors:** Annie YS Lau, Kalman Piper, Desmond Bokor, Paige Martin, Victor SL Lau, Enrico Coiera

**Affiliations:** ^1^ Centre for Health Informatics Australian Institute of Health Innovation Macquarie University Sydney Australia; ^2^ Department of Orthopaedic Surgery Faculty of Medicine & Health Sciences Macquarie University Sydney Australia

**Keywords:** mobile application, mobile health, personal health record, patients, health services, medical informatics, surgery, orthopedics, shoulder, rotator cuff, rehabilitation

## Abstract

**Background:**

Translating research into practice, especially the implementation of digital health technologies in routine care, is increasingly important. Yet, there are few studies examining the challenges of implementing patient-facing digital technologies in health care settings.

**Objective:**

The aim of this study was to report challenges experienced when implementing mobile apps for patients to support their postsurgical rehabilitation in an orthopedic setting.

**Methods:**

A mobile app was tailored to the needs of patients undergoing rotator cuff repair. A 30-min usability session and a 12-week feasibility study were conducted with patients to evaluate the app in routine care. Implementation records (observation reports, issues log, and email correspondence) explored factors that hindered or facilitated patient acceptance. Interviews with clinicians explored factors that influenced app integration in routine care.

**Results:**

Participant completion was low (47%, 9/19). Factors that affected patient acceptance included digital literacy, health status, information technology (IT) infrastructure at home, privacy concerns, time limitations, the role of a caregiver, inconsistencies in instruction received from clinicians and the app, and app advice not reflective of patient progress over time. Factors that negatively influenced app integration in routine care included competing demands among clinicians, IT infrastructure in health care settings, identifying the right time to introduce the app to patients, user interface complexity for older patients, lack of coordination among multidisciplinary clinicians, and technical issues with app installation.

**Conclusions:**

Three insights were identified for mobile app implementation in routine care: (1) apps for patients need to reflect their journey over time and in particular, postoperative apps ought to be introduced as part of preoperative care with opportunities for patients to learn and adopt the app during their postoperative journey; (2) strategies to address digital literacy issues among patients and clinicians are essential; and (3) impact of the app on patient outcomes and clinician workflow needs to be communicated, monitored, and reviewed. Lastly, digital health interventions should supplement but not replace patient interaction with clinicians.

## Introduction

### Background

The use of health and wellness applications have grown rapidly over the past years [1], including the implementation of digital health technologies in routine care. Such technology has the potential to provide ongoing personalized care for patients. However, the elements that contribute to an effective app, as well as the best ways to integrate *patient-facing* apps in routine care, are relatively unexplored. In parallel, the lack of evidence for apps’ efficacy and effectiveness continues to be a key barrier to mainstream adoption of mobile apps in routine care [1,2].

To date, the majority of literature focuses on implementing clinician-facing digital technologies in health care settings [3,4], with few studies examining the challenges of implementing patient-facing digital technologies in routine care [2]. This study reports on the challenges experienced when implementing mobile apps for patients in routine care, focusing on supporting patients undergoing rotator cuff repair in an orthopedic setting.

### Rotator Cuff Repair

The shoulder joint is a ball-and-socket joint between the scapula (socket) and the humerus (ball). The rotator cuff is a group of four tendons that connect the muscles attached to the scapula to the humerus. The function of the rotator cuff is to rotate the ball in the socket and therefore, move the arm.

Tears of the rotator cuff are a common cause of shoulder pain and upper limb weakness [5]. The tears of the rotator cuff commonly occur with upper limb injury or with age-related degeneration [6]. Often, this injury can be treated nonsurgically; however, depending on the patient, the tear, and severity of the injury, surgical repair may be required [5].

To achieve the best results from surgery, patients should adhere to a strict postoperative rehabilitation protocol to prevent retearing of the tendon and regain maximum shoulder function (Multimedia Appendix 1). This protocol is a local guideline developed by consensus between surgeons at Macquarie University Hospital (MUH) participating in this study. It includes wearing a sling, completing daily exercises, limiting shoulder use, and attending physical therapy. A recent study has identified a positive relationship between poor patient adherence to the rehabilitation protocol and an increased rate of rotator cuff retear during the first 12 postoperative weeks [7]. One of the reasons for poor adherence with the rehabilitation protocol is the tendency for patients to diminish the importance of the protocol over time, as visits to their surgeon become less frequent and their level of pain decreases.

### Study Focus

To improve patient adherence with the rehabilitation protocol, a mobile app using the Healthy.me platform was developed for patient use. Full details of the Healthy.me platform are described elsewhere [8-13]. The rationale for using an app is the convenience of having rehabilitation information (including exercise videos and contact information) easily accessible via a mobile phone and the ability of the app to encourage adherence to the rehabilitation protocol outside of visits with health care professionals.

The aim of this feasibility study is to examine factors that facilitate or hinder the implementation of a patient-facing app in routine clinical care following rotator cuff surgery.

## Methods

### Study Design

A 30-min usability session and a 12-week study were conducted with patients undergoing rotator cuff repair surgery to evaluate the usability, feasibility, and acceptance of the app to support the patient’s postoperative rehabilitation. A mixed-methods approach was used to incorporate the collection of quantitative app usage data, qualitative data through feedback from patients and clinicians, as well as implementation records taken by researchers during the study.

This evaluation was performed on patients attending for surgical treatment at MUH, Sydney, New South Wales (Australia), which is a private teaching and tertiary referral hospital. Personnel involved in the study included orthopedic surgeons, practice and ward nurses, health informaticians, software engineers, and research and administrative staff. Ethics approval was obtained from the Macquarie University Human Research Ethics Committee.

Patients booked for rotator cuff repair at MUH were eligible to participate in this study if they were in the age range of 40 to 65 years, English-speaking, in possession of an Internet-enabled iPhone or Android mobile phone, and intended to undergo surgical treatment for rotator cuff repair.

All participants received standard postoperative care. Participants were required to complete a 10- to 15-min questionnaire at their first preoperative visit and at their routine visits 2 weeks, 6 weeks, and 12 weeks postoperatively. Participants could comment further on a voluntary basis via email or telephone during the 12-week study period.

### Patient Recruitment

Eligible patients suitable for the study were initially recruited by the surgeon, practice nurse, or a research team member during the patients’ preoperative consultation at the orthopedic clinic. Patients could also be recruited by the ward nurse during their recovery in the ward after surgery.

Patients suitable for the study were initially recruited by the surgeon, practice nurse, or a research team member during the patients’ preoperative consultation at the orthopedic clinic. Patients could also be recruited by the ward nurse during their recovery in the ward after surgery.

Participants provided written informed consent. They were advised that they could cease app use at any time and return to standard care involving regular outpatient clinic visits. They were also given an email address and a mobile phone number to a research team member for queries, issues, or comments during the 12-week period.

### App Development and Features

A steering group with 5 representatives from orthopedics, consumer informatics, and software development was formed and met over 3 months for 2 hours every fortnight to codesign the app, formulate the study design, and compose educational content for patients. An internal usability study with 10 individuals was conducted with all major usability issues addressed before patient recruitment. Three meetings were also held with all clinicians involved to refine ways to introduce the app to patients before study commencement.

**Figure 1:**
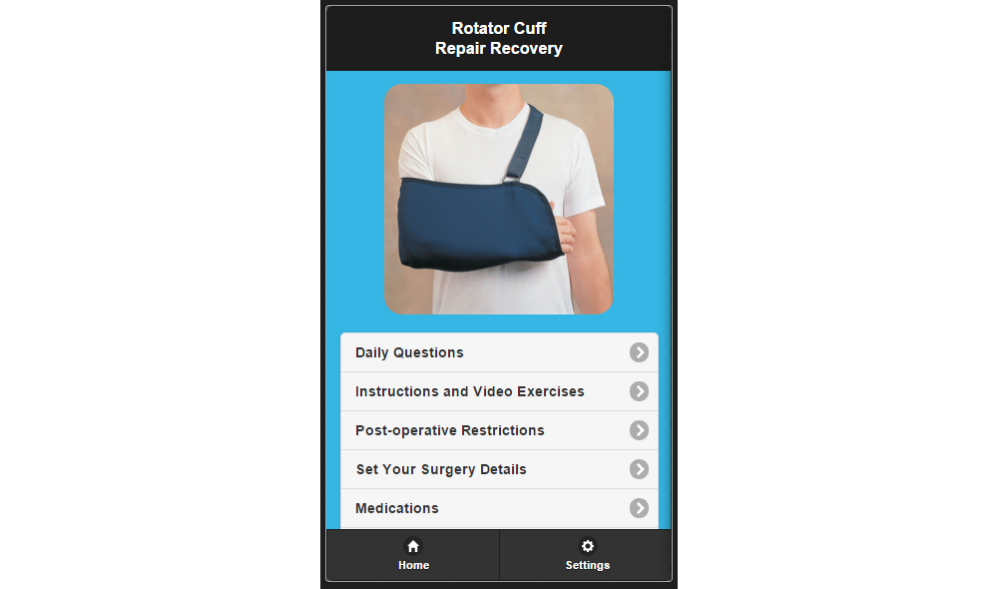
Home page of the app developed to support patient postoperative rehabilitation after rotator cuff repair.

A mobile app was developed that contains information on the postoperative rehabilitation program. It contains (1) postoperative rehabilitation exercise videos; (2) important information and restrictions at different stages of the recovery; (3) contact information of the surgeon, practice nurse, and the research team; and (4) a *pillbox* for patients to record their medications and dosage. Figure 1 shows a screenshot of the home page of the app. Full details of the app development process are outlined in Multimedia Appendix 2.

To improve adherence with the rehabilitation protocol, patients were encouraged to complete a 3-min questionnaire daily within the app (Multimedia Appendix 3). The questionnaire was designed to address common issues relevant to the participant’s stage of postoperative recovery (Multimedia Appendix 1). Participants were sent a weekly SMS text message (short message service, SMS) reminder to complete the questionnaire (Multimedia Appendix 1).

Whereas the app is not available for public use, the app was installed on the mobile phones of participants in this feasibility study using the TestFlight platform on iPhone operating system (iOS, Apple Inc) devices. Participants with an Android device were provided with an URL, where participants could download the app directly from Google Play Store, following email validation by the research team.

### Data Collection and Analysis

#### App Usage Data

Patient app usage was assessed using a system log that recorded time of app access, app features used, and the duration and frequency of use for each app feature. Descriptive statistics were computed for the usage data.

#### Questionnaire Data

The Western Ontario Rotator Cuff Index is a standardized and validated questionnaire that quantifies pain, analgesic usage, and quality of life specific to rotator cuff disease [14,15]. The questionnaire was completed by the participants preoperatively and at their routine 2-week, 6-week, and 12-week postoperative visits. Clinical outcomes of these data are not reported here.

#### Implementation Records

Implementation records (ie, field notes recorded by researchers that detail the implementation process of the app) were collected during the study. These include participant observation reports, issues log, and email correspondence with participants. For participants recruited by the research team, observation reports were made in accordance to a predefined template (Multimedia Appendix 1) during the usability session. These reports contained details of the usability session, researcher observations of the participant (eg, body language and who else was there), and issues that had facilitated or hindered participants’ use of the app. An *issues log* (eg, technical problems with the app or telephone conversations with participants) was also maintained by the research team during the study.

#### Semistructured Interviews

At the completion of the 12-week study, a member independent of the app development team conducted a semistructured interview with the clinicians involved to explore key issues that emerged from the implementation records. An interview schedule was developed in consultation with the research team (Multimedia Appendix 1). Responses from early interviews were used to refine the schedule for later interviews [16]. No patients were available for poststudy interview because of lack of availability or interest.

#### Mapping of Patient Workflow

Before clinician interviews, a series of cross-functional flowcharts showing a workflow were drawn for patients requiring rotator cuff repair at MUH, representing the interactions between four main settings: the patient’s home, general practitioner (GP), orthopedic clinic, and hospital (including ward nurse and physiotherapist). Standard flowchart symbols were used [17] (Multimedia Appendix 1). The initial flowcharts were drafted by the research team based on their observations, and interviews with clinicians served to verify the patient workflow steps.

#### Qualitative Data Analysis

For the qualitative data analysis, audiotapes were transcribed verbatim. Two members (AL and VL) read through all interview transcripts and implementation records independently, following the constant comparative method and thematic analysis [18,19]. An initial thematic framework was developed from a sample of transcript and record data, with VL coding the remaining data according to the framework, with no new themes or revisions. AL then reexamined the themes and supporting quotes, and results were discussed with all the authors. Any disagreement was resolved via group discussion and consensus. Rigor was addressed by coding according to a comprehensive framework; an iterative process of constant comparison between framework and data; and discussion of themes with all the authors [20]. Quotes are reported with no alterations.

## Results

### Patient Characteristics

Participant completion rate was low (47%, 9/19). Participants were in the age range of 42 to 67 years (mean=55.4 years, standard deviation [SD]=8.6 years). One participant (aged 67 years) was above the age eligibility criteria but was included in the feasibility study because of his enthusiasm to participate in the study. Patient demographic data are shown in Multimedia Appendix 1. (Note: participants are included in the study if they have provided consent, and completed most of the questionnaires or installed the app).

### App Usage Statistics

Eight out of 9 participants installed the mobile app. System log showed that the mean duration of app usage was 46.9 days (SD=24.5 days, median=42.5 days, interquartile range [IQR]=29 days). A total of seven app features were monitored, namely, home page, daily questionnaire, exercise videos, rehabilitation information, surgery details, contact information, and pillbox. Access frequencies for each feature are outlined in Multimedia Appendix 1. For those who have installed the app (n=8), most (6/8) have used all features of the app but at different levels of frequency (Multimedia Appendix 1). Across participants, apart from accessing the home page (mean of 121 times per participant during the 12 weeks, SD=49, median=134.5, IQR=55), completion of daily questionnaires on rehabilitation adherence (mean=45, SD=24, median=40, IQR=26.5) was the primary activity. The journeys that contained exercise videos (mean=10, SD=5, median=11, IQR=4.5) and rehabilitation information (mean=10, SD=7, median=8, IQR=5) were the next most accessed features.

### Workflow of Patients Requiring Rotator Cuff Surgery

**Figure 2:**
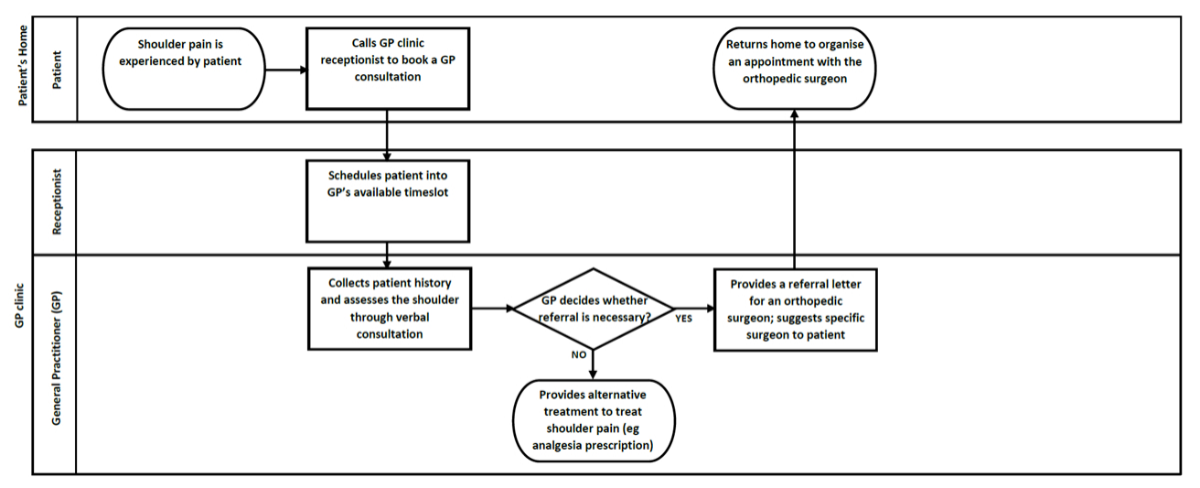
Patient workflow for a general practitioner (GP) referral to an orthopedic surgeon.

**Figure 3:**
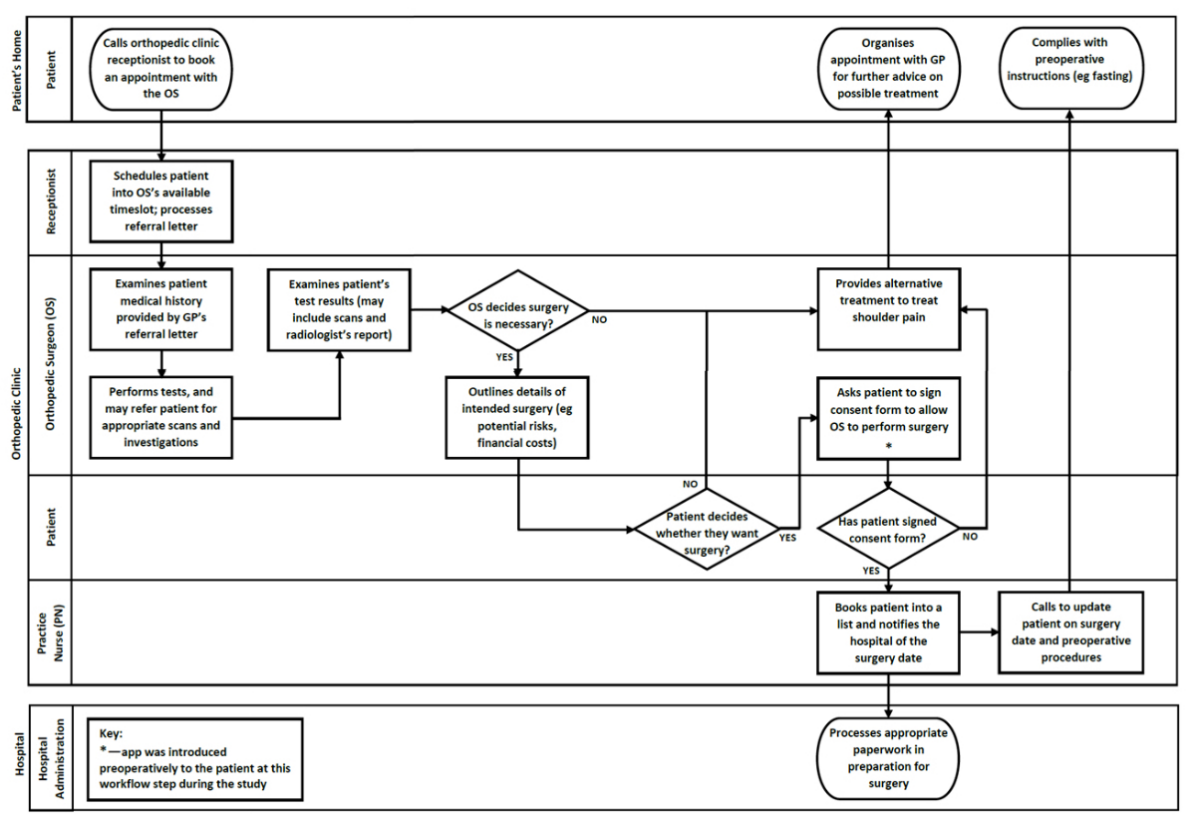
Patient workflow for the preoperative procedures required before surgery.

**Figure 4:**
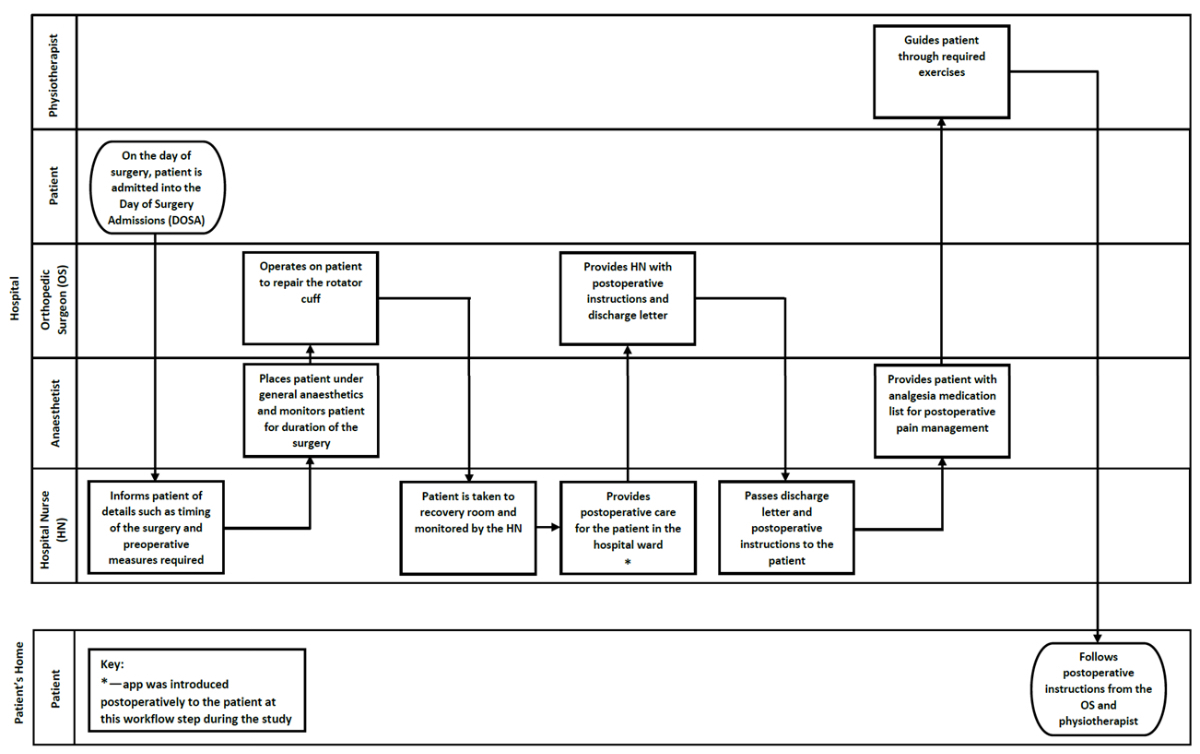
Patient workflow during a rotator cuff repair surgical procedure.

**Figure 5:**
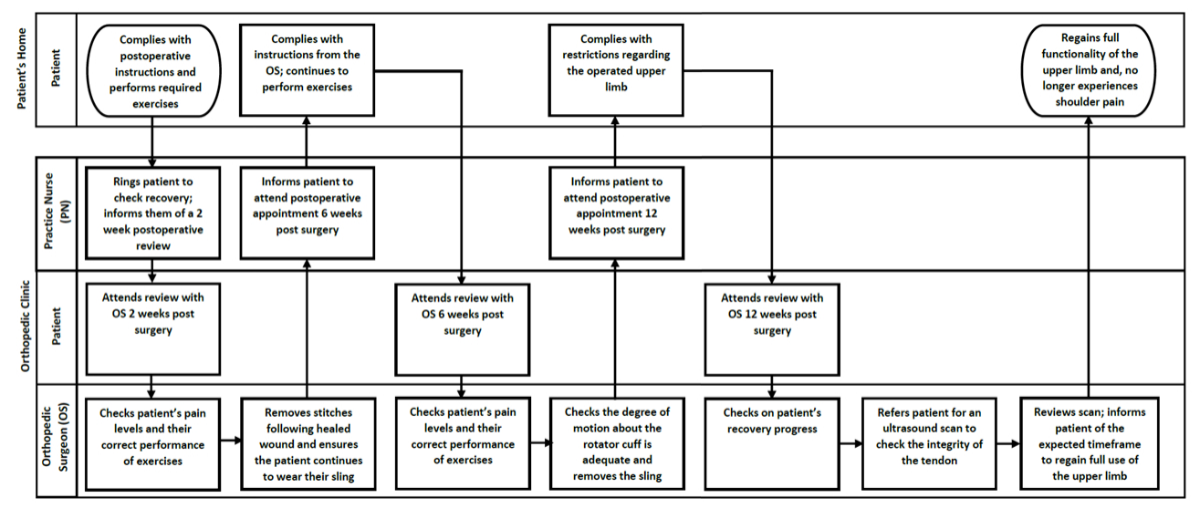
Patient workflow for postoperative reviews with an orthopaedic surgeon.

Figures 1-4 detail the main steps of patient workflow required for a rotator cuff repair and the subsequent rehabilitation procedures at MUH. These flowcharts describe a set of chronological stages for patients undertaking rotator cuff repair. Legends for flowchart symbols are listed in Multimedia Appendix 1. Figures 2-5 describe each of these stages.

### Patient Perceptions

Patient feedback was obtained from three sources: observation reports at the preoperative meeting, issues log, and email correspondence collected during the study. Issues reported by patients or recorded by researchers are detailed in Textbox 1.

Patient perspectives on the role of the app in their postoperative rehabilitation are illustrated below.

Questionnaires regarding rotator cuff recovery need to more accurately reflect the patient’s journey:

I felt that if the questions changed to reflect the recovery milestones I had reached, then the app would be more relevant.

Participant ID 3, Age 42, Female

I thought the questions were quite good for the first six weeks but needed to be changed for the second six weeks as they are less relevant and this affects the frequency of filling out the questions.

Participant ID 1, Age 67, Male

The inability of the app’s questionnaires to relate to more specific scenarios:

I think there is a fundamental question missing from this questionnaire, namely, is the operated arm dominant arm? This would greatly affect the answers to many of these questions.

Participant ID 1, Age 67, Male

The questions about pain might be more useful if they included a question about pain when exercising. I have very little pain when undertaking normal activities but during and after exercises I have a much higher level of pain.

Participant ID 1, Age 67, Male

Sleeping has also contributed some level of pain and stiffness as I have reverted from sleeping propped up on my back to sleeping on my non operated side (I use to always sleep on my operated side).

Participant ID 1, Age 67, Male

### Clinician Perceptions

Clinician feedback was obtained from two sources: implementation records during the study and semistructured interview at poststudy. Issues reported by clinicians or recorded by researchers are reported in Textbox 2.

Clinicians’ perspectives on factors that affected patient acceptance and app integration in routine care are outlined below. Some clinicians have also provided suggestions on remedies:

Lack of coordination among multidisciplinary clinicians about new changes from the app:

The rehab program we brought in for the app was an agreed standard program that I’m happy with but most physios elsewhere have got my old rehab schedule not the new rehab schedules. So it’s a matter of actively changing across the board places where they’re going to go and formalising the fact that we’ve changed our rehab program so I think it’s just a communication issue.

Clinician ID 4

Deficient digital literacy experienced among patients:

If it’s the elderly, it’s negative [responses] because half of them don’t even know how to use the phone. The smartphone.

Clinician ID 2

Firstly, the demographic, 40-65 has a higher proportion of “tech-ludites” There are people who use a smartphone but don’t use apps...Secondly, there are people who think they are good with apps and they were daunted...And there were a number of people who...once were shown the app, go...too many steps. If you come back and do the study in 20 years’ time, everyone will be okay with it.

Clinician ID 4

Time investment involved through daily consultation with the app:

Some said that they were too busy, that they didn’t want to. That was probably the main [reason].

Clinician ID 5

**Patient issues regarding the mobile app.**

1. Population variation in digital health literacy

- Patients not remembering iPhone operating system **(**iOS **)** passwords when installing app.
- Patients forgetting app password when attempting to log in.
- Patients unfamiliar with Wi-Fi setup at home and expressed concern on how to access the app at home.
- Patients with high digital health literacy did not express any difficulties with the app.
- One patient expressed preference to install the app on their iPad, which was not on their person, resulted in a verbal account of instructions. That patient could complete installation successfully at home.
- One patient expressed unfamiliarity with the app download process, which raised questions on whether the use of mobile apps for such patients is appropriate.

2. Patient state and their health status (eg, pain, fatigue, comorbidities, and their concerns)

- Patients were disengaged during the usability study, possibly because of pain (confirmed by some patients verbally and observed through body language) or low digital health literacy, leading to unfamiliarity with app.
- Patients’ preexisting orthopedic conditions or multiple injuries excluded their eligibility to use the app as the exercises were designed only for people with rotator cuff repair.
- Patients were unaware of the extent of postoperative restrictions until informed by the app.
- Patients seemed engaged throughout the session but started becoming impatient with the number of questionnaires.
- One patient expressed concern regarding how his ability to work will be affected (as he operates machinery at work).

3. App needs to more accurately reflect patient recovery journey

Patients expressed that the app needs to be more reflective of patient recovery journey, such as questions changed to reflect recovery milestones, provision of advice for specific scenarios, and that some restrictions need to be relaxed in certain circumstances as patients progress over time.

4. Privacy concerns over data collection

Patients express concerns over privacy and sharing of information with institutions, such as insurance and workers’ compensation (eg, WorkCover).

5. Role of caregivers

- Patients with low mobile phone literacy depend on caregivers to install the app. In one scenario, caregiver also did not display adequate digital literacy to comfortably install the app (eg, forgetting Apple ID and iOS password).
- Patient reliance on caregiver leads to participant withdrawal from study as caregiver becomes unavailable to look after patient.

6. Time limitations

- In-person meetings were carried out immediately after patients attended their first meeting with their surgeon, and some patients did not have the time to stay for an additional 20 to 30 min to meet with researchers about the study.
- One recruited patient pulled out because of time investment required being too much at the usability study.

7. Credibility of app content

- Patients asked if exercise information was approved by surgeons.

**Clinician issues regarding the mobile app.**

1. Lack of coordination among multidisciplinary clinicians

- Surgeons have their own rehabilitation protocol. Designing this app was also a way to consolidate differences in approaches and attitudes among surgeons in standardizing a postoperative rehabilitation protocol.
- However, some physiotherapists were not aware of the new rehabilitation protocol and thus, provided instructions to patients that were not consistent with those embedded in the app.
- Similarly, one patient reported discrepancies between the app and clinician instructions at hospital discharge.

2. Deficient digital literacy skills among clinicians

- Some clinicians noted that a self-perceived lack of confidence and skills in using new technologies may have hindered their willingness to introduce the app to patients.

3. Demanding workload and competing demands

- Clinicians are often too busy (or have forgotten) to introduce the app to patients.

Introducing the app during the preoperative stages of a rotator cuff surgical patient:

I think the big issue would be adequate pre-op engagement and instruction.

Clinician ID 4

Pre-operatively, because you need all that information pre-operatively. You need them to remember their pain levels and all that sort of thing and their discomfort. So it definitely would have to be started pre-operatively.

Clinician ID 5

New app features to closely reflect patient recovery journey:

The trouble is that it runs for six weeks and the patients are doing the same exercises for six weeks and after two weeks, they know what they’re doing. Maybe we could look at… they get prompted every day for the first week or two but then for the next four weeks, maybe they get contacted less.

Clinician ID 3

What I really think would be good is to have some sort of generated referral [reminder] when it’s time to have the scans done, I think that would be good...And maybe a booking system online with the app so then they’re given a date and they can choose the time that suits them so that it’s an automated [process].

Clinician ID 5

Suggestions on ways to educate patients on how to use the app:

One thing that probably we didn’t think about in the app is a practice app. Something that says let’s pretend you’ve had something, let’s pretend you’re [at] day 5, these are the questions that you’ll be asked so that they get to play with it without it being recorded as part of their management but purely as their education.

Clinician ID 4

When you download the app, download an instructional video that whenever in doubt, it can say this is what you got to do...

Clinician ID 4

### Other Issues

Other issues emerged regarding the organizational setting are reported in Textbox 3. Technological issues with the app are outlined in Textbox 4.

**Organizational issues regarding the mobile app.**

1. Information technology infrastructure

- Failure to connect to the local Wi-Fi at outpatient clinic or in hospital.

2. Timing of introducing the app to patients

- Determining the optimum timing, persons of contact, and patient recruitment logistics across multiple health care settings is not simple.
- The need to constantly remind clinicians to offer patients to partake in the study.
- (For inpatients recruited postoperatively) Without knowing the app’s existence before surgery, patients may have the propensity to decline automatically to participate, impeding patient recruitment rate.

3. Training clinicians on digital literacy and informing on impact is important

- Strategies to improve digital literacy and inform frontline clinicians on ways to integrate the app into routine care (and the subsequent impact on workflow and workload) are important.

**Technical issues regarding the mobile app.**

1. Problems with app installation

- Installation process for iPhone operating system **(**iOS **)** TestFlight has many steps that can be confusing for patients, possibly dissuading further involvement.
- App installation depends on receiving an email, with patients not necessarily linking their email account to their device. (In addition, there is the inability to customize TestFlight emails or the email address they are sent from. These emails could be categorized as spam and may cause confusion as they originate from a name and email address unknown to the user).

2. Problems with app distribution

- Issues with TestFlight hindering app distribution. For example, app expires on TestFlight after 60 days, where users must update the app. Users receive a notification detailing app expiration, which may have misled them to think that the study has ended.
- Distribution issues may reflect poorly on app developers, despite this issue being unable to be resolved from the app developer team.

3. Pre- and postimplementation support

- Troubleshooting of issues is more difficult to occur without an onsite technician.
- Some recruited patients did not respond to initial emails or secondary follow-up emails.
- Time spent providing technical support to and liaising with participants is significant, and appropriate resources should be allocated for this.

## Discussion

### Principal Findings

This feasibility study offers insights into the implementation of a mobile app for patients in an orthopedic setting. Our mixed-methods approach has identified factors that affected patient acceptance of the app ranging from patient-related factors (such as digital literacy and health status), contextual factors (eg, IT infrastructure at home, time limitations, and the role of their caregiver), personal concerns (eg, privacy over information sharing), to other factors (such as inconsistencies in instruction received from clinicians and the app, and app advice not reflective of patient progress over time). In parallel, factors that negatively influenced app integration in routine care include clinician-related factors (eg, competing demands, heavy workload, digital literacy, and lack of coordination among multidisciplinary team), organizational factors (eg, IT infrastructure in health care settings and optimal timing of introducing the app to patients), and issues related to the app (eg, user interface complexity for older patients and technical issues with app installation).

Whereas this study focused on rotator cuff repair, barriers experienced from patients, clinicians, and health care organizations (Textboxes 1-4) are highly likely applicable to other health care settings when implementing patient-facing apps in routine care.

### Comparison With Prior Work

The use of mobile health technology as an intervention to provide patient care is becoming increasingly common [18,19]. They have been used to support chronic disease management such as diabetes, cardiovascular, chronic lung diseases (ie, chronic obstructive pulmonary disease and asthma), mental health, and osteoarthritis [21]. Whereas these technologies involve a wide range of functionality to support patient self-management (such as to inform, instruct, record, display, guide, remind or alert, and communicate) [21], its application in the surgical setting remains limited.

At the time of writing, we are only aware of the mobile app developed by Semple et al to monitor patient recovery at home after surgery [22-24]. Semple et al demonstrated successful acceptance of the app in a feasibility study, with all 65 participants who were undergoing either breast reconstruction or orthopedic surgery completing the study. However, the examined cohort of Semple et al was of a relatively young age, and their associated familiarity with technology could be a contributing factor to their willingness to use mobile apps.

Our study, however, only targeted patients from an older age bracket of 40 to 65 years and experienced a lower study completion rate (47%). It has been acknowledged that a reduced usage of technology by the older population can be attributed to a variety of reasons, including but not limited to a deficient understanding of the benefits of mobile apps to provide care, reluctance to gain digital literacy skills, and physical impairments leading to a lacking confidence in navigating through app features [25]. By addressing these issues, app uptake by the older age group could potentially be improved in future studies.

### Implications for Implementing Patient-Facing Apps in Routine Care

We identified three key challenges that impeded app uptake and integration during routine care and proposed suggestions to address them. These challenges may also be factors that contributed to the low participation or completion rate in this study.

#### Implementation Ought to Be Patient-Centric at All Times

Our findings indicated that advice from the app was not relevant to patients’ recovery journey on some occasions. Apps should provide advice that relates to specific patient scenarios, recovery milestones, and individual progress, intelligently adapting to the patient’s changing condition over time. They should also be designed and implemented with the patient in mind, considering that patients could be experiencing pain, fatigue, comorbidities, and concerns that could affect their decision to use (or not use) the app at any time point during their treatment journey.

Our study also found that identifying an optimal time to introduce the app to patients in routine care is not straightforward. Patients may not have been ready to participate when the study was first introduced at the preoperative stage. The lead up to surgery is often a challenging time for patients, as they are anxious regarding the surgery and often are making significant adjustments to their regular work and home routines to accommodate the time off required for the surgery. However, patients should still have an opportunity to learn about the app during the postoperative stage, even when they have declined the opportunity to use the app earlier in their treatment journey.

To encourage uptake, apps designed for postoperative recovery ought to be introduced as part of preoperative care, where there are opportunities for patients to learn and adopt the app during their postoperative journey. Starting the recruitment process earlier in the patient journey, as well as having multiple points for patients to learn (or remind them) about the app postoperatively, could potentially improve participant uptake.

#### Implementation Should Be Digitally Inclusive for Patients and Clinicians

Our findings revealed that some patients may not have the digital health literacy necessary to use the app as a form of postoperative care, although there were some exceptions. Digital literacy concerns among *patients* should be addressed appropriately and accordingly, with adequate resources and support. Ownership of a mobile phone device is not a sufficient eligibility criteria, as participants may not be able to use the device to the full extent. There was a wide range of digital literacy competencies among participants in this study, ranging from those who only use a mobile phone for answering calls, texting, and have never used an app, to those who regularly use complex apps and felt that the app used in this study was too simple and did not address all their needs. The question of how to balance the high expectations of users with high digital literacy, and those struggling with low digital literacy, is an important issue in app design.

Our study also found that digital literacy concerns among clinicians need to be addressed. There were occasions when clinicians did not feel confident enough to introduce the app to patients because of their self-perceived lack of familiarity with apps. For example, some clinicians felt uneasy helping patients set up the app or addressing any technical concerns during app installation. Strategies to improve digital literacy among frontline clinicians, to help them tackle common problems expressed by patients during app installation and usage, and ways to incorporate the app into their work routine are of utmost importance.

Overall, the level of time and resources required in providing digital literacy support to patients and clinicians can be intensive. A *practice app* may assist those with low digital literacy become familiar with the app. To achieve implementation success of digital health technology in routine care, a sufficient budget to support patients and clinicians with their digital literacy concerns is necessary.

#### Implementation Needs to Be Communicated, Monitored, and Reviewed

Our study indicated that not all clinicians involved in patient care were aware of the app, resulting in inconsistencies in patient instructions received from some clinicians and the app. Communication with all clinicians involved is paramount. All parties need to be informed on how the app will impact on their clinic workflow, responsibilities, and patient communication. The overall impact of the app on patient outcomes and the flow-on effects on staff workload needs to be communicated, monitored, and reviewed. Ultimately, digital technologies should supplement and not replace patient interaction with clinicians.

Our study also found that clinicians were often too busy (or have forgotten) to introduce the app to patients because of their heavy workload and competing demands. The rotator cuff patient workflow is more complex than was originally anticipated (as indicated in Figures 2-5). There are many unanswered questions regarding the effects of introducing *patient-facing* technology into this complex workflow and how these technologies may affect clinician workload, patient-clinician interaction, and patient expectations. For example, what role should these technologies have in routine care? How, when, and where should they be introduced to patients? How do they affect clinician workload and workflow? Do they introduce any unintended consequences? Whereas the literature for implementing *clinician-facing* technologies is increasing, more empirical and theoretical guidance is required for implementing *patient-facing* technologies in routine care and personal settings.

### Limitations

Several limitations need to be noted:

- Participant completion rate (47%) was low, which limited study generalizability. However, we have identified a range of factors that hindered the implementation of patient-facing apps in routine care, which is the major focus of this report.
- Participants were only recruited from MUH (an academic private hospital), where socioeconomic status of patients is likely higher than the general community. It has been previously reported that individuals with a higher socioeconomic status also have higher digital literacy rates [26]. However, we experienced a range of digital literacy competencies among our participants. Challenges regarding digital literacy could be significantly larger for studies conducted in public hospitals primarily treating patients with lower socioeconomic status.
- Using alternative platforms to distribute the app (such as TestFlight) may have hindered uptake rates, as most participants were not familiar with this app download process. When working with participants who may have digital literacy concerns, using traditional platforms for app distribution rather than alternative platforms may improve uptake rates.
- Although an analysis of the app usage system log revealed quantitative evidence of the most frequently used app features, this may not be reflective of the patients’ app experience. Unfortunately, no patient participants in this study were available for follow-up interviews. The app may not meet patients’ changing needs and expectations during their recovery, which may be factors contributing to the low uptake. Future studies could consider using a theoretical framework to guide study implementation. These frameworks include (but are not limited to) the Exploration, Preparation, Implementation, and Sustainment (EPIS) framework [27]; Consolidated Framework for Implementation Research (CFIR) [28]; Promoting Action on Research Implementation in Health Services (PARiHS) framework [29]; and the Reach, Effectiveness, Adoption, Implementation, and Maintenance (RE-AIM) framework [30]. Studies should also measure patients’ adherence to the app with strategies in place to improve participant follow-up.
- The rehabilitation protocol that the app was designed upon was specific to patients with rotator cuff tears undergoing surgery, which may have limited the intake of participants, as well as the generalizability of the app to other conditions. A wider range of exercise protocols could be implemented in the app so that it complements a larger pool of patients recovering from different injuries and surgical procedures. Future studies could also consider recording patient adherence to in-person rehabilitation programs and examine whether it is a contributing factor to app adherence.
- In addition, we did not recruit family members or caregivers to be participants of the study. As indicated in our study, caregivers have an important role to play in surgical patients’ postoperative recovery; future studies could consider recruiting patients’ caregivers as study participants to elicit their views.

### Conclusions

The potential of mobile apps to support patient care is increasingly recognized, but they are still not routinely recommended by clinicians or integrated as part of standard care [19]. In this feasibility study, many challenges were identified, and we have emphasized three insights when implementing *patient-facing* technologies in routine care:

1. the importance for implementation to remain *patient-centric* at all times
2. to be inclusive of patients and clinicians of varying levels of digital literacy
3. the impact of the technology on patients and clinician workflow needs to be communicated, monitored, and reviewed.

Ultimately, digital health technology should supplement and not replace patient interaction with clinicians. Consumer, clinician, and service provider involvement are vital if mobile health is to fulfill its potential.

The science of implementing *patient-facing* technologies remains underexplored. Yet, the challenges in implementation are often not reported nor perceived important in academic literature [31]. With the emergence of next generation personal health technologies (eg, wearables, sensors, and medical devices) and their increasing popularity in the general population, further research is required to guide the implementation of *patient-facing* technologies across health care and personal settings to maximize their potential and prevent harm.

## Appendix

Multimedia Appendix 1 Supplementary tables.

Multimedia Appendix 2 App development and features.

Multimedia Appendix 3 Daily patient questionnaire.

## Abbreviations

CFIR: Consolidated Framework for Implementation Research
EPIS: Exploration, Preparation, Implementation, and Sustainment framework
GP: general practitioner
IQR: interquartile range
IT: information technology
MUH: Macquarie University Hospital
PARiHS: Promoting Action on Research Implementation in Health Services framework
RE-AIM: Reach, Effectiveness, Adoption, Implementation, and Maintenance framework
SD: standard deviation

## References

[1] (2015). IMS Institute for Healthcare Informatics.

[2] Steinhubl SR, Muse ED, Topol EJ (2015). The emerging field of mobile health. Sci Transl Med.

[3] Glasgow RE, Vinson C, Chambers D, Khoury MJ, Kaplan RM, Hunter C (2012). National Institutes of Health approaches to dissemination and implementation science: current and future directions. Am J Public Health.

[4] Lehmann CU, Altuwaijri MM, Li YC, Ball MJ, Haux R (2008). Translational research in medical informatics or from theory to practice. A call for an applied informatics journal. Methods Inf Med.

[5] Clement ND, Nie YX, McBirnie JM (2012). Management of degenerative rotator cuff tears: a review and treatment strategy. Sports Med Arthrosc Rehabil Ther Technol.

[6] Neer 2nd CS (1983). Impingement lesions. Clin Orthop Relat Res.

[7] Ahmad S, Haber M, Bokor DJ (2015). The influence of intraoperative factors and postoperative rehabilitation compliance on the integrity of the rotator cuff after arthroscopic repair. J Shoulder Elbow Surg.

[8] Lau AY, Sintchenko V, Crimmins J, Magrabi F, Gallego B, Coiera E (2012). Impact of a web-based personally controlled health management system on influenza vaccination and health services utilization rates: a randomized controlled trial. J Am Med Inform Assoc.

[9] Arguel A, Lau AY, Dennis S, Liaw ST, Coiera E (2013). An internet intervention to improve asthma management: rationale and protocol of a randomized controlled trial. JMIR Res Protoc.

[10] Lau AY, Dunn AG, Mortimer N, Gallagher A, Proudfoot J, Andrews A, Liaw ST, Crimmins J, Arguel A, Coiera E (2013). Social and self-reflective use of a Web-based personally controlled health management system. J Med Internet Res.

[11] Lau AY, Proudfoot J, Andrews A, Liaw ST, Crimmins J, Arguel A, Coiera E (2013). Which bundles of features in a Web-based personally controlled health management system are associated with consumer help-seeking behaviors for physical and emotional well-being?. J Med Internet Res.

[12] Mortimer NJ, Rhee J, Guy R, Hayen A, Lau AY (2015). A web-based personally controlled health management system increases sexually transmitted infection screening rates in young people: a randomized controlled trial. J Am Med Inform Assoc.

[13] Lau AY, Arguel A, Dennis S, Liaw ST, Coiera E (2015). “Why Didn't it Work?” Lessons from a randomized controlled trial of a web-based personally controlled health management system for adults with asthma. J Med Internet Res.

[14] de Witte PB, Henseler JF, Nagels J, Vliet Vlieland TP, Nelissen RG (2012). The Western Ontario rotator cuff index in rotator cuff disease patients: a comprehensive reliability and responsiveness validation study. Am J Sports Med.

[15] Kirkley A, Alvarez C, Griffin S (2003). The development and evaluation of a disease-specific quality-of-life questionnaire for disorders of the rotator cuff: The Western Ontario Rotator Cuff Index. Clin J Sport Med.

[16] Jootun D, McGhee G, Marland GR (2009). Reflexivity: promoting rigour in qualitative research. Nurs Stand.

[17] American National Standards Institute (1970). Flowchart Symbols and Their Usage in Information Processing.

[18] Boulos MN, Brewer AC, Karimkhani C, Buller DB, Dellavalle RP (2014). Mobile medical and health apps: state of the art, concerns, regulatory control and certification. Online J Public Health Inform.

[19] Singh K, Drouin K, Newmark LP, Lee J, Faxvaag A, Rozenblum R, Pabo EA, Landman A, Klinger E, Bates DW (2016). Many mobile health apps target high-need, high-cost populations, but gaps remain. Health Aff (Millwood).

[20] Barbour RS (2001). Checklists for improving rigour in qualitative research: a case of the tail wagging the dog?. BMJ.

[21] Laranjo L, Lau A, Oldenburg B, Gabarron E, O’Neill A, Chan S (2015). Sax Institute.

[22] Armstrong KA, Semple JL, Coyte PC (2014). Replacing ambulatory surgical follow-up visits with mobile app home monitoring: modeling cost-effective scenarios. J Med Internet Res.

[23] Armstrong KA, Coyte PC, Bhatia RS, Semple JL (2015). The effect of mobile app home monitoring on number of in-person visits following ambulatory surgery: protocol for a randomized controlled trial. JMIR Res Protoc.

[24] Semple JL, Sharpe S, Murnaghan ML, Theodoropoulos J, Metcalfe KA (2015). Using a mobile app for monitoring post-operative quality of recovery of patients at home: a feasibility study. JMIR Mhealth Uhealth.

[25] Holzinger A, Searle G, Nischelwitzer A (2007). On Some Aspects of Improving Mobile Applications for the Elderly.

[26] Neter E, Brainin E (2012). eHealth literacy: extending the digital divide to the realm of health information. J Med Internet Res.

[27] Aarons GA, Hurlburt M, Horwitz SM (2011). Advancing a conceptual model of evidence-based practice implementation in public service sectors. Adm Policy Ment Health.

[28] Damschroder LJ, Aron DC, Keith RE, Kirsh SR, Alexander JA, Lowery JC (2009). Fostering implementation of health services research findings into practice: a consolidated framework for advancing implementation science. Implement Sci.

[29] (2011). National Collaborating Centre for Methods and Tools.

[30] King DK, Glasgow RE, Leeman-Castillo B (2010). Reaiming RE-AIM: using the model to plan, implement, and evaluate the effects of environmental change approaches to enhancing population health. Am J Public Health.

[31] Bennett-Levy J, Singer J, DuBois S, Hyde K (2017). Translating e-mental health into practice: what are the barriers and enablers to e-mental health implementation by Aboriginal and Torres Strait Islander health professionals?. J Med Internet Res.

