# Supplementary material for: Challenges During Implementation of a Patient-Facing Mobile App for Surgical Rehabilitation: Feasibility Study
Source: JMIR Hum Factors. 2017 Dec 7;4(4):e31. doi: 10.2196/humanfactors.8096 (PMC5740262; doi:10.2196/humanfactors.8096)
Supplement: Multimedia Appendix 1 [file humanfactors_v4i4e31_app1.pdf]

Multimedia Appendix 1

Table A. Rehabilitation protocol for a rotator cuff repair

Stage	Standard Procedure
Post-operative review by surgeon at 2 weeks	• Questionnaire<br>• Examination findings<br>• Removal of stiches if the wound has healed<br>• Surgeon checks correct performance of exercises by patient
Post-operative review by surgeon at 6 weeks	• Questionnaire<br>• Examination findings<br>• Remove sling if an adequate range of motion is experienced<br>• Referral to physiotherapy; patient is to attend appointment at least once a fortnight, preferably once a week<br>• Surgeon checks correct performance of exercises by patient
Post-operative review by surgeon at 12 weeks	• Questionnaire<br>• Examination findings<br>• Ultrasound scan to determine integrity of tendon

Table B. Timing of weekly SMS messages sent to patients during rehabilitation

Timing	SMS Message
7pm,<br>1 day after surgery	Wearing a sling can be frustrating, but please ensure that you keep your sling on 24 hours a day, even in the shower. Also, don't forget to complete your questionnaire daily.
7pm,<br>8 days after surgery	Are you in a lot of pain despite taking your pain meds? If so, consider contacting your surgeon's practice. Also, don't forget to complete your questionnaire daily.
7pm,<br>15 days after surgery	Are you having trouble with your exercises? You can always consult the 'Exercise Instructions & Videos' page in Healthy.me for detailed information on how to perform them. Also, don't forget to complete your questionnaire daily.
7pm,<br>21 days after surgery	If you drink tea or coffee, make sure that you don't handle the kettle or the milk bottle with your operated arm, as picking up anything heavier than a cup of tea can re-injure your shoulder. Also, don't forget to complete your questionnaire daily.
7pm,<br>28 days after surgery	Not being able to drive is very inconvenient, but please do not attempt to as you could risk re-injury if you use your operated arm to drive. Also, don't forget to complete your questionnaire daily.
7pm,<br>35 days after surgery	Have you attended your 6 week follow up with your surgeon yet? It should be scheduled in this week. Also, don't forget to complete your questionnaire this week.
7pm,<br>42 days after surgery	Your arm might be feeling better, but please make sure that you avoid using your operated arm to do anything, as even small movements such as using a computer mouse can increase your risk of re-injury. Also, don't forget to complete your questionnaire this week.
7pm,<br>49 days after surgery	Are you still taking pain meds? If you are, but you're in little to no pain, consider reducing your dosage. Also, don't forget to complete your questionnaire this week.
7pm,<br>56 days after surgery	How are you going with the exercise program prescribed by your physio? It is important to ensure that you are performing these as frequently as prescribed. Also, don't forget to complete your questionnaire this week.
7pm,<br>63 days after surgery	As you ease back into your usual activities, ensure that you avoid movements that involve lifting your arm away from your body or above shoulder height, such as combing your hair, as this can cause re-injury. Also, don't forget to complete your questionnaire this week.
7pm,<br>70 days after surgery	Is your pain almost gone? If so, that's fantastic, but please ensure that you do not use your operated arm to tuck your shirt in at the back, or put it behind your back for any reason at all, as this can cause re-injury. Also, don't forget to complete your questionnaire this week.
7pm,<br>77 days after surgery	Have you attended your 12 week follow up with your surgeon yet? It should be scheduled in this week. Also, don't forget to complete your questionnaire this week.

Table C. Template of observation reports made of patient during the usability session

Template Type	Details
Sole Patient Observation Template	**Patient Name:**<br>**Date:**<br>**Surgery Date:**<br>**Interactions between people**<br>● People present and their roles:<br>● Who spoke with who about what, & how many times?<br>○ E.g. Patient → Researcher: App questionnaire<br>○ E.g. Patient → Researcher: Information privacy<br>**Context**<br>● People involved with patient<br>● Who is the surgeon?<br>● Who is the practice contact?<br>**Patient State**<br>● In pain?<br>● Engaged or disinterested?<br>● Attitude towards app?<br>● Likely to use app?<br>● Attitude towards rehab protocol?<br>● Likely to adhere to rehab protocol?<br>● Concerns raised?<br>● Questions asked?<br>● eHealth literacy?<br>**User issues encountered during usability session:**<br>**Any other issues encountered/things of note during session:**
Caregiver-Patient Relationship Observation Template	**Patient Name:**<br>**Caregiver Name:**<br>**Date:**<br>**Surgery Date:**<br>**Caregiver relationship to patient:**<br>**Interactions between people**<br>● People present and their roles:<br>● Who spoke with who about what, & how many times?<br>○ E.g. Patient → Caregiver: Pain relief<br>○ E.g. Patient → Researcher: Information privacy<br>**Context**<br>● People involved with patient<br>● Who completed pre-op questionnaires?<br>● Whose phone is app installed on?<br>● Whose details are registered in app?<br>○ Phone:<br>○ Email:<br>○ Name:<br>● Who is more likely to use app?<br>○ To what extent?<br>● Who is the surgeon?<br>● Who is the practice contact?<br>**Patient State**<br>● In pain?<br>● Engaged or disinterested?<br>● Attitude towards app?<br>● Likely to use app?<br>● Attitude towards caregiver?<br>● Attitude towards rehab protocol?<br>● Likely to adhere to rehab protocol?<br>● Concerns raised?<br>● Questions asked?<br>● eHealth literacy?

	**Caregiver State**<br>● Engaged or disinterested?<br>● Attitude towards app?<br>● Attitude towards patient?<br>● Likely to use app/encourage patient use of app?<br>● Likely to encourage patient adherence to rehab protocol?<br>● Concerns raised?<br>● Questions asked?<br>● eHealth literacy?<br><br>**User issues encountered during usability session:**<br>**Observations about patient/caregiver use of app:**<br>**Any other issues encountered/things of note during session:**

Table D. Template of finalised semi-structured interview questions with clinicians

Template Type	Details
Question set for orthopaedic surgeon	Q. A problem encountered during this study was patients having a different set of exercises for rehabilitation than those embedded in the app, since their surgery wasn't a straight rotator cuff repair. As such, these patients couldn't be recruited for this study. What suggestions do you have for improving the app's design to account for this?<br><br>Q. A planned diagram for the manuscript maps out the steps that a patient must go through to receive rotator cuff surgery (*draft diagrams are shown*). Can you verify that the steps shown are correct?<br><br>Q. When a patient comes in for a post-operative appointment, what is the usual procedure? How do you think the implementation of the app into the system would change this?<br><br>Q. Do you have any suggestions on how to improve the app? Which features did you find useful? Which features did you find not so useful?<br><br>Q. If we were to run this study again, what changes or improvements would you suggest (e.g. in terms of the app, recruitment procedures, workflow etc.)<br><br>Q. Are there any other comments/thoughts/suggestions that you would like to make?
Question set for practice nurse or ward nurse	Q. Can you describe your experience of patient responses when you were recruiting them? How many patients did you approach and how many agreed to participate?<br>Q. For patients who agreed to participate, were they enthusiastic about the study? Did these patients give any reasons for wanting to participate?<br>Q. If patients didn't want to be involved, did they give any reasons?<br>Q. Were there any barriers to the recruitment process that you experienced?<br>Q. A planned diagram for the manuscript maps out the steps that a patient must go through to receive rotator cuff surgery (*draft diagrams are shown*). Can you verify that the steps shown are correct?<br>Q. What steps can we take to improve patient recruitment for future studies? For example, at which point in the patient workflow do you think we could have introduced the study to patients in order to maximise patient recruitment?<br>Q. Do you have any suggestions on how to improve the app? Which features did you find useful? Which features did you find not so useful?<br>Q. If we were to run this study again, what changes or improvements would you suggest (e.g. in terms of the app, recruitment, workflow etc.)<br>Q. Are there any other comments/thoughts/suggestions that you would like to make?

Table E: Definition of standard flowchart symbols

Shape	Meaning
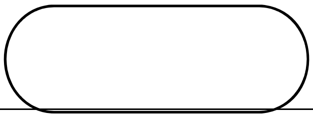	Represents the start or end of a process.
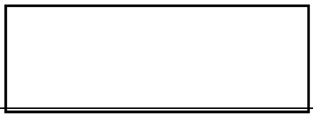	Represents a step or action which occurs within a process.
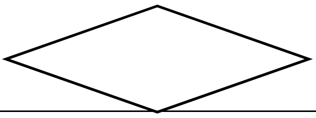	Represents a decision which needs to be made. Labelled with a question which is answered by either ‘Yes’ or ‘No’ and the direction of the process is determined by the answer to the question.
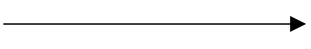	Represents the interaction between different individuals within and/or between settings. The direction of the workflow progresses from the tail to the head of the arrow.

Table F: Characteristics of patients (N=9) who used the Healthy.me app during the study

Patient ID	Age (years)	Gender	Used apps before?	Length of Shoulder Pain (months)	Occupation	Number of times app was opened
1	67 [a]	Male	Yes	9	Retired	42
2	n/a [b]					64
3	42	Female	Yes	12	Technical Learning Consultant	97
4	55	Male	Yes	7	Truck Driver	52
5	62	Female	Yes	5	Administration	52
6	45	Male	Yes	12	Doctor Psychiatrist	14
7	53	Female	Yes	12	Shop Assistant	72
8	57	Female	No	2.5	Registered Nurse	40
9	62	Female	No	12	n/a [b]	n/a [c]

[a] Participant was above the age eligibility criteria, but was still included in the feasibility study

[b] Participant did not fill in appropriate sections of the pre-operative questionnaire and thus did not provide us with this identifying information

[c] Participant did not install app and only filled in the necessary questionnaires

Table G: Frequency of access by patients (N=8) for each app feature during the study

	Number of Times Accessed over the 12-week Feasibility study, n (%)								
	**ID 1**	**ID 2**	**ID 3**	**ID 4**	**ID 5**	**ID 6**	**ID 7**	**ID 8**	**Mean (SD)**
Home Page	129 (68.3)	106 (54.6)	156 (57.4)	140 (62.5)	156 (68.4)	16 (53.3)	165 (61.8)	96 (57.1)	121 (49)
Daily Questionnaire	34 (18.0)	54 (27.8)	92 (33.8)	38 (17.0)	42 (18.4)	14 (46.7)	60 (22.5)	27 (16.1)	45 (24)
Exercise Videos	8 (4.2)	11 (5.7)	5 (2.9)	18 (8.0)	11 (4.8)	0 (0)	11 (4.1)	14 (8.3)	10 (5)
Rehabilitation information	8 (4.2)	5 (2.6)	9 (3.3)	13 (5.8)	9 (3.9)	0 (0)	10 (3.7)	24 (14.3)	10 (7)
Surgery Details	7 (3.7)	9 (4.6)	3 (1.1)	5 (2.2)	5 (2.2)	0 (0)	8 (3.0)	3 (1.8)	5 (3)
Pillbox	0 (0)	3 (1.5)	3 (1.1)	4 (1.8)	4 (1.8)	0 (0)	8 (3.0)	3 (1.8)	3 (3)
Contact information	3 (1.6)	6 (3.1)	1 (0.4)	6 (2.7)	1 (0.4)	0 (0)	5 (1.9)	1 (0.6)	3 (2)
