# Supplementary material for: Challenges During Implementation of a Patient-Facing Mobile App for Surgical Rehabilitation: Feasibility Study
Source: JMIR Hum Factors. 2017 Dec 7;4(4):e31. doi: 10.2196/humanfactors.8096 (PMC5740262; doi:10.2196/humanfactors.8096)
Supplement: Multimedia Appendix 2 [file humanfactors_v4i4e31_app2.pdf]

Multimedia Appendix 2: App Development and Features

Using the Healthy.me platform, a mobile app was developed that contains information on the post-operative rehabilitation program, inclusive of videos demonstrating specific exercises. In addition, a SMS was sent to patients weekly, prompting them to complete a 3-minute daily questionnaire within the app, which helps them adhere to rehabilitation protocol by addressing common issues relevant to the participant's stage of post-operative recovery (Multimedia Appendix 1 Table B).

The app also contains contact information of their surgeon, practice nurse, and the research team, and a pillbox for patients to record their medications and adherence.

Three RC "journeys" were developed, providing pragmatic material for patients about postoperative exercises and restrictions. The concept of a journey, which is a key component of the Healthy.me platform is described elsewhere [8-13]. One example of such a journey allows patients, in conjunction with a nurse, to set specific details about their surgery (e.g. name and contact details of surgeon, physiotherapist, surgery date, hospital, and post-operative medications).
