# Supplementary material for: Challenges During Implementation of a Patient-Facing Mobile App for Surgical Rehabilitation: Feasibility Study
Source: JMIR Hum Factors. 2017 Dec 7;4(4):e31. doi: 10.2196/humanfactors.8096 (PMC5740262; doi:10.2196/humanfactors.8096)
Supplement: Multimedia Appendix 3 [file humanfactors_v4i4e31_app3.pdf]

Multimedia Appendix 3: Daily Patient Questionnaire

1. How many times have you done your exercises today?
   a. 0
   b. 1-2
   c. 3
   d. 3+
2. Please rate your pain last night on a scale of 1-10
3. Please rate your pain today on a scale of 1-10
4. Which pain medications have you taken in the last 24 hours (patients may select multiple options)
   - ☐ None
   - ☐ Paracetamol (Panadol, Panamex etc)
   - ☐ Paracetamol + Codeine (Panadiene Forte)
   - ☐ Anti-Inflammatory (Voltaren, Mobic, Celebrex)
   - ☐ Oxycodone Slow Release (Targin, OxyContin)
   - ☐ Oxycodone (Endone)
   - ☐ Pregabalin (Lyrica)
   - ☐ Other (free text entry)
5. Apart from when doing your exercises, have you worn your sling all day today, including in the shower?
   a. Yes
   b. No
6. Have you used your operated arm to brush your teeth[1] today?
   a. Yes
   b. No

[1] The action patents are questioned about is selected randomly from a bank of actions including teeth brushing, using a knife or computer mouse, driving the car, etc.
